# The Association between Serum Follistatin-like Proteins and Cardiovascular Diseases: A Systematic Review and Meta-analysis

**DOI:** 10.2174/011573403X304195240715103930

**Published:** 2024-07-18

**Authors:** Amir Mahmoud Ahmadzadeh, Rozita Khodashahi, Mahmoud Mohamadzadeh Shabestari, Mohsen Aliakbarian, Amirhossein Sahebkar, Mohammad-Hassan Arjmand

**Affiliations:** 1 Transplant Research Center, Clinical Research Institute, Mashhad University of Medical Sciences, Mashhad, Iran;; 2 Department of Radiology, School of Medicine, Mashhad University of Medical Sciences, Mashhad, Iran;; 3 Clinical Research Development Unit, Imam Reza Hospital, Faculty of Medicine, Mashhad University of Medical Sciences, Mashhad, Iran;; 4 Department of Cardiology, Emam Reza Hospital, School of Medicine, Mashhad University of Medical Sciences, Mashhad, Iran;; 5 Surgical Oncology Research Center, Mashhad University of Medical Sciences, Mashhad, Iran;; 6 Center for Global Health Research, Saveetha Medical College and Hospitals, Saveetha Institute of Medical and Technical Sciences, Saveetha University, Chennai, India;; 7 Biotechnology Research Center, Pharmaceutical Technology Institute, Mashhad University of Medical Sciences, Mashhad, Iran;; 8 Applied Biomedical Research Center, Mashhad University of Medical Sciences, Mashhad, Iran

**Keywords:** Follistatin-like protein, cardiovascular disease, adipomyokine, databases, biomarker, quantitative analysis

## Abstract

**Background:**

Follistatin-like proteins (FSTLs) are adipomyokines secreted by adipocytes and myocytes. Previous studies have reported an increase in circulating FSTL1 levels in response to cardiovascular injuries. In this study, we conducted a systematic review and meta-analysis to assess the association between circulating FSTLs and Cardiovascular Diseases (CVDs).

**Methods:**

We performed a comprehensive literature search using PubMed, Web of Science, Scopus, and Embase databases. After screening the articles, we selected eligible studies, extracted relevant data, and calculated the pooled Standardized Mean Difference (SMD). We also conducted a sensitivity analysis to identify sources of heterogeneity and assessed publication bias.

**Results:**

Among the 577 articles initially retrieved, we included 5 studies comprising a total of 941 cases with CVDs and 446 controls. All included studies measured FSTL1 levels. The pooled SMD analysis revealed a significant difference in circulating FSTL1 levels between subjects with CVDs and control groups (SMD = 0.853, 95% CI = 0.158-1.548, *P* = 0.016). Heterogeneity was primarily attributed to a single study that measured FSTL1 levels in heart failure patients with preserved ejection fraction. No publication bias was observed.

**Conclusion:**

Our findings demonstrate significantly higher levels of FSTL1 in patients with CVD compared to control subjects. This suggests that FSTL1 may have potential as a diagnostic and prognostic biomarker in CVDs. However, further well-designed studies are needed to validate its clinical utility.

## 1 INTRODUCTION

Cardiovascular Diseases (CVDs), such as ischemic heart disease, heart failure, stroke, peripheral artery disease, and other heart and vascular pathologies, are the leading cause of mortality and morbidity worldwide [1, 2]. Approximately 17.5 million people die from CVDs each year [3]. Despite advancements in biomedical technology, the search for new biomarkers associated with biological pathways remains a crucial area of focus for improving diagnostic and therapeutic strategies. Recently, there has been growing interest in the potential role of adipomyokines as biomarkers in CVDs. Adipose tissue and muscles play important roles in fat storage and metabolic regulation, releasing bioactive substances that influence various physiological and biological processes [4-6]. Adipomyokines, a group of bioactive molecules released by both adipose tissue and muscles, act as autocrine/paracrine mediators and regulate molecular mechanisms [7, 8].

Follistatin-like proteins (FSTLs) belong to the secreted protein acidic and rich in cysteine (SPARC) protein family and are classified as adipomyokines [9, 10]. There are five types of FSTLs, including FSTL1 to FSTL5. FSTL homologues exert autocrine and paracrine activities, modulating intracellular signaling pathways. The levels of FSTLs have been found to be altered in various pathological conditions, including inflammatory diseases [11, 12], cancer progression [13, 14], and CVDs [15, 16]. A study by Kizer *et al.* reported a significant association between FSTL3 and the incidence of heart failure [17]. Additionally, previous research has shown a significant association between FSTL1 levels and insulin resistance and physical activity [9]. Yamazaki *et al.* demonstrated that plasma FSTL1 levels were associated with the severity of coronary artery disease, suggesting a potential role of FSTL1 in the pathogenesis of coronary artery atherosclerosis [18]. Conversely, Oshim *et al.* identified FSTL1 as a cardiac protective factor, showing that administration of recombinant FSTL1 to mice reduced myocardial infarct size and cardiomyocyte apoptosis following ischemia/reperfusion injury [19]. Therefore, the aim of this study is to conduct a systematic review and meta-analysis to provide evidence-based results regarding the association between circulating FSTLs and CVDs.

## 2 METHOD

This study was conducted in accordance with the Preferred Reporting Items for Systematic Reviews and Meta-Analyses (PRISMA) statement.

### 2.1 Search Strategy

A comprehensive literature search was performed in four electronic databases, including PubMed, Web of Science, Scopus, and Embase, to select relevant studies until the end of September 2023. The terms used in the search are described in the supplementary file **1**. The MeSH terms were also integrated into the search keywords.

### 2.2 Inclusion and Exclusion Criteria

Studies that reported serum/plasma levels of FSTLs in subjects with and without CVDs (comprising diseases related to the heart and its vascular structures, cerebrovascular diseases, and peripheral artery diseases) and provided sufficient data for estimating Standardized Mean Difference (SMD) were included. Due to the significance of comprehensive data analysis, we sent emails to the corresponding authors of the congress abstracts, stating that we believed their data could be potentially included in our study. Exclusion criteria were as follows: 1) irrelevant studies; 2) original studies without enough data or control group; 3) review articles, letters, editorials, case reports/series; 4) congress abstracts if the corresponding authors did not reply to our emails; 5) studies that focused on the FSTLs expression in heart tissue rather than serum/plasma; 6) animal/*in vitro*/*ex vivo* studies; and 7) not English articles.

### 2.3 Data Extraction and Quality Assessment

RK and MA independently screened all the retrieved papers based on title/abstract. Any disagreements were resolved by a third reviewer (MMS). Two authors (AMA and MHA) independently read the full text of the selected articles and extracted relevant data including the name of first author and year of publication, country of origin, study design, CVD type, sample size, report of consecutive sampling, gender, age, FSTL type, method of measurement, serum/plasma FSTL level, inclusion and exclusion criteria, BMI, and number of patients with the history of hypertension, diabetes mellitus, smoking, or dyslipidemia. Quality assessment of each study was performed using the Newcastle-Ottawa Scale (NOS) [20].

### 2.4 Statistical Analysis

Outcomes were reported as SMD with 95% CI based on the mean and SD extracted from the included studies to assess the difference in circulating FSTL levels in subjects with and without CVDs. Heterogeneity was calculated using the Cochran Q statistics and inconsistency index (I^2^) among the included studies. Due to the heterogeneity in study characteristics (including country, study design, CVD type, sample size, and measurement method), we used a random-effects model. Sensitivity analysis was also performed by the sequential exclusion of individual trials to find out the causes of heterogeneity. For the calculation of SMD, we converted the median and Interquartile Range (IQR) to mean and standard deviation [21]. Furthermore, we used Repeating Cochrane's formula to combine groups of means and standard deviations into a single group [22]. Evidence of publication bias was calculated by applying funnel plots and Egger’s linear regression test. Duval and Tweedie’s trim and fill test was also performed to find the number of trimmed studies and their effects on the pooled SMD. Meta-analysis was performed using Comprehensive Meta-Analysis (CMA) software version 3 (Biostat, Englewood, NJ), and statistical significance was defined as *p*-value < 0.05.

## 3 RESULTS

### 3.1 Literature Search

The initial electronic database search identified 850 results, of which 577 articles remained after removing duplicates. After the first assessment of titles and abstracts, 493 papers were excluded, and 33 papers remained for full-text evaluation (two studies were in the form of abstracts [23, 24]. To get full data, we sent emails to the corresponding authors, but we did not receive any response to include them in the study). After full-text evaluation, 5 studies were considered appropriate and were included for the estimation of pooled SMD [18, 22, 25-27]. A summary of the detailed process of inclusion is shown in Fig. (**1**).

### 3.2 Study Characteristics

The characteristics of the selected papers are shown in Table **1**. All of the included articles were published between 2009 and 2023. A total of 623 cases and 321 controls were included in this meta-analysis. Two studies were conducted in the USA [25, 26], two in Japan [18, 22], and one in Germany [27]. In all the included studies, the type of FSTL measured was FSTL1 [18, 22, 25-27]. The method of FSTL measurement was ELISA in all studies except one paper that used western blotting [26].

### 3.3 Quantitative Analysis

**Fig. (2):**
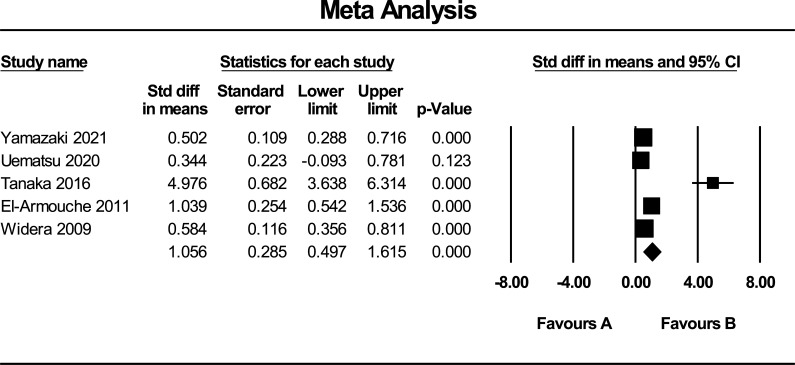
Pooled SMD (random-effect model) of circulating FSTL1. SMD: standard mean difference, FSTL1: follistatin-like protein 1.

**Fig. (3):**
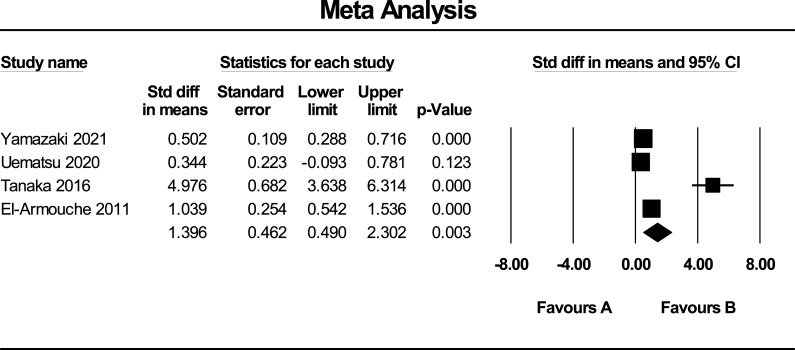
Forest plot (random-effects model) of circulating FSTL1 in chronic phase of CVDs. FSTL1: follistatin-like protein 1, CVD: cardiovascular disease.

Data regarding the mean and SD of circulating FSTL1 levels of the 5 included papers were pooled to evaluate the summary SMD as an estimate of the difference in circulating FSTL1 in subjects with and without CVDs. Overall, our meta-results indicated a significant difference in the circulating FSTL1 levels between subjects with CVDs and the control groups (SMD = 1.06 and 95% CI = 0.497-1.615, *P* < 0.001) (Fig. **2**). Based on the measurement of FSTL1 levels in the acute and chronic phases of the CVDs, we excluded one study from the analysis due to the assessment of FSTL1 level in the acute phase of the disease [27] and the result yielded a pooled SMD of 1.396 (95% CI = 0.490-2.302, *P* = 0.003) (Fig. **3**).

### 3.4 Heterogeneity Analysis

**Fig. (4):**
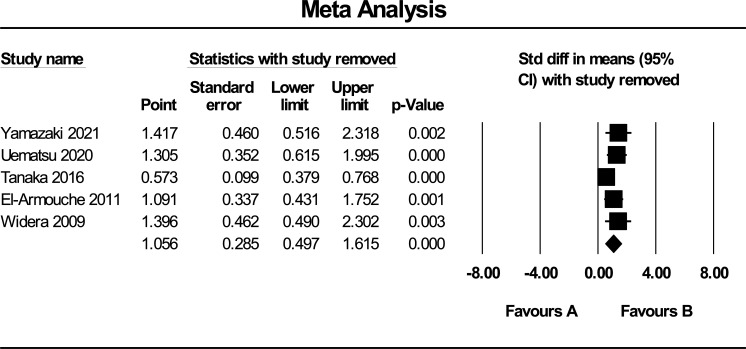
Sensitivity analysis of the included studies.

Due to the limited number of eligible articles, we did not perform subgroup analysis. Sensitivity analysis was performed to explore the impact of a single study or other factors that may influence the pooled SMD (Fig. **4**). The results revealed that one study (Tanaka *et al.* [25]) was responsible for the high heterogeneity. This might be justified by measuring circulating FSTL1 levels in subjects with preserved ejection fraction rather than individuals with reduced ejection fraction. Pooled SMD analysis, after excluding the mentioned study, led to a significant decrease in heterogeneity (I^2^ = 37.8, *P* = 0.18).

### 3.5 Publication Bias

Begg’s funnel plots were drawn, and Egger’s regression asymmetry test was carried out to evaluate the publication bias (Fig. **5A**). The results revealed no publication bias (*P* = 0.08). Duval and Tweedie’s trim and fill test revealed one trimmed study. This method yielded a pooled SMD of 1.37, which shows a greater difference in the SMD between the groups after considering the one trimmed study (Fig. **5B**).

## 4 DISCUSSION

Based on our comprehensive analysis, this meta-analysis represents the first attempt to assess the difference in circulating FSTL levels between patients with and without CVDs. The findings of this study demonstrate a significant association between increased FSTL1 levels and CVDs. By pooling the SMDs from the five included studies, we observed a higher pooled SMD in the group with CVDs compared to the group without CVDs. These results suggest that FSTL1 can potentially serve as a biomarker for CVDs.

Adipomyokines, which are secreted by both adipose tissue and muscles, are bioactive molecules that modulate various intracellular molecular pathways [28]. FSTLs, as a subset of adipomyokines, have been implicated in diverse biological processes, including wound healing, inflammation, and fibrosis [29]. In recent years, several studies have investigated the roles of FSTLs in the cardiovascular system. The concentration of circulating FSTL1 was found to be elevated in the cardiac tissue of patients with heart failure and was correlated with cardiac function during recovery [30]. FSTL1 binds to the receptor disco-interacting protein 2 homolog A, activating the phosphatidylinositol 3-kinase (PI3K)/protein kinase B (Akt) pathway. This leads to an upregulation of the mammalian target of rapamycin (mTOR) activity, which exerts anti-apoptotic and proliferative effects [31]. *In vivo* studies have shown that FSTL1 overexpression attenuates myocardial apoptosis and reduces infarct size following ischemia/reperfusion injury. This cardioprotective effect is mediated through the promotion of Akt and extracellular signal-regulated kinase (ERK) activity [19]. FSTL1 has also been implicated in promoting angiogenesis and revascularization in ischemic conditions by activating the Akt/endothelial nitric oxide synthase (eNOS) signaling pathway [32]. Additionally, FSTL1 can act as an inhibitor of activins, members of the TGF-β family that contribute to the pathogenesis of heart failure [30].

During the meta-analysis, we observed a high degree of heterogeneity among the included studies (I2 = 91.34, *P* < 0.001). To investigate the source of this heterogeneity, we conducted a sensitivity analysis and identified one study [25] as the potential cause (after excluding this study: I2 = 37.8, *P* = 0.18). In this particular study, the FSTL1 levels were significantly higher in the case group compared to the control group. This could be attributed to the measurement of FSTL1 levels in heart failure patients with preserved ejection fraction as the case group. FSTL1 is mainly expressed by the cardiomyocytes, myocardium endothelial cells, and smooth muscle cells of the vessels [33]. The higher competence and viability of cardiomyocytes in CVD patients with preserved ejection fraction could potentially account for the observed difference in the SMD.

In addition to the high heterogeneity observed in the included studies, our study had several other limitations. The number of relevant studies available in the literature was limited, and we were only able to include five studies in our meta-analysis. Furthermore, due to the lack of sufficient studies, we could not estimate the SMD for other FSTL homologues (*e.g*., FSTL3). Additionally, conducting subgroup analyses was not feasible due to the limited number of studies available for inclusion. These limitations should be taken into consideration when interpreting the results of our meta-analysis.

## CONCLUSION

In conclusion, our meta-analysis revealed a significant association between higher levels of circulating FSTL1 and CVDs in comparison to individuals without CVDs. However, it is important to acknowledge the limitations of our study, including the high heterogeneity among the included studies and the small number of studies available for analysis. Therefore, further research is needed to strengthen the evidence and provide more robust results. Future studies could explore the levels of other FSTL homologs in individuals with and without CVDs to assess their potential role as biomarkers. Additionally, investigating the differences in FSTL levels across different categories of CVDs could provide valuable insights.

## AUTHORS’ CONTRIBUTIONS

A.M.A., R.K., M.M.S., M.A., A.S., and M-H.A. contributed to the research design and implementation, as well as the data analysis and manuscript writing.

## Figures and Tables

**Fig. (1):**
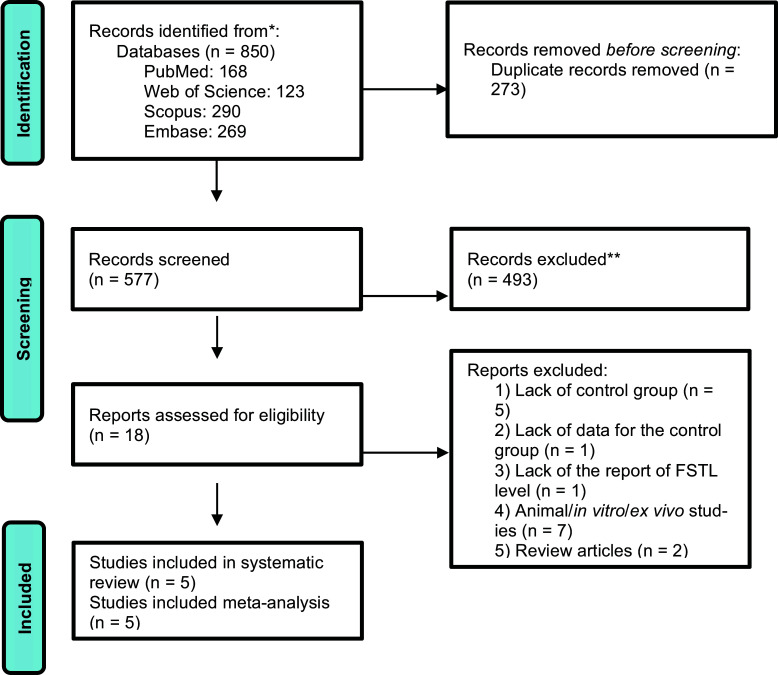
Flow diagram of the study selection process.

**Fig. (5A):**
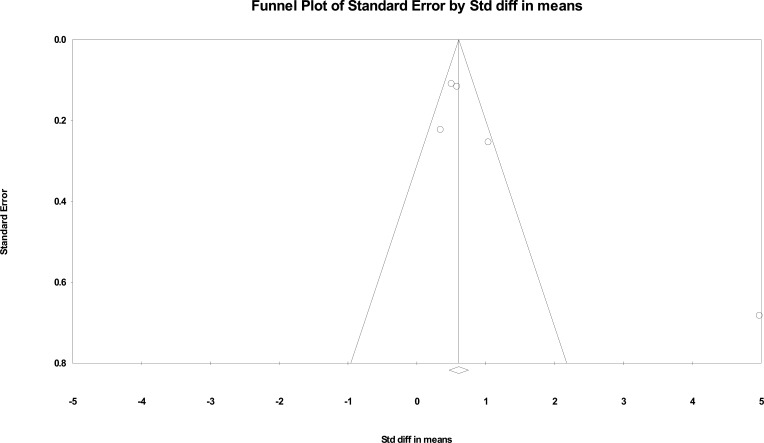
Begg’s funnel plot with pseudo 95% CI.

**Fig. (5B):**
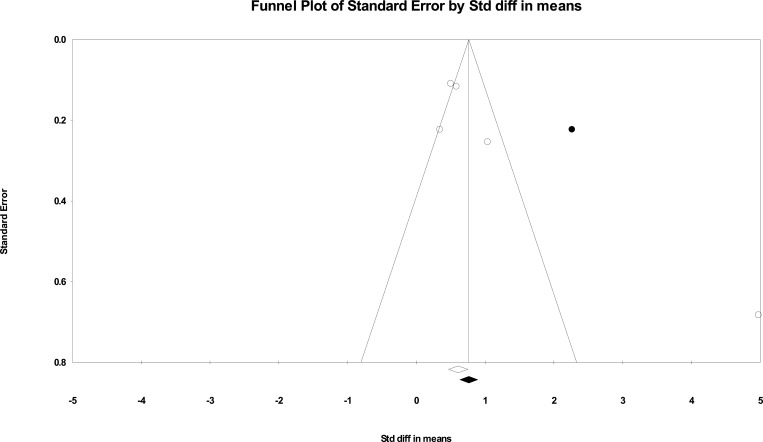
The result of Duval and Tweedie’s trim and fill test (one study was trimmed).

**Table 1:** Characteristics of studies included in the meta‐analysis.

**No.**	**Author & Year**	**Country**	**Study Design**	**Study Date**	**CVD type**	**Sample Type**	**Sample Size (Case/Control)**	**Report of Consecutive Sampling**	**Male/Female (Case/Control)**	**Age (year, mean ± SD) (Case/Control)**	**FSTL type**	**Method of Measurement**	**FSTL Level in Serum (ng/ml) (mean ± SD)**		**Quality**
													**Control**	**Case**	
1	Yamazaki 2021	Japan	Prospective	NR	CAD	Plasma	196/154	Yes	149/47 88/66	70 ± 9 64 ± 12	FSTL1	ELISA	3.65 ± 1.56	4.15 ± 1.79	8
2	Uematsu 2020	Japan	Retrospective	July 2006-November 2015	MI	Plasma	93/26	Yes	75/18 21/5	66.7 ± 13.3 65.1 ± 8.6	FSTL1	ELISA	0.97 ± 1.47	2.147 ± 3.78	5
3	Tanaka 2016	USA	Retrospective	NR	HF	Serum	32/8	No	45/55 NR	65 ± 2 NR	FSLT1	ELISA	95.6 ± 16.0	167.2 ± 14.0	7
4	El-Armouche 2011	USA	Prospective	2001-2004	HF	Serum	86/21	No	52/34 12/9	60 ± 13 58 ± 8	FSTL1	Western Blotting	12.7 ± 5	19.8 ± 7.2	7
5	Widera 2009	Germany	Prospective	August 2007-September 2008	ACS	Serum	216/120	Yes	180/36 60/60	65 ± 11.85 44.3 ± 35.5	FSTL1	ELISA	8.91 ± 12.92	13.50 ± 1.93	9

**Abbreviations:** ACS: acute coronary syndrome, CAD: coronary artery disease, HF: heart failure, MI: myocardial infarction, PAD: peripheral arterial disease, NR: not report.

## Data Availability

The data and supportive information are available within the article.

## LIST OF ABBREVIATIONS

FSTLs: Follistatin-like Proteins
CVDs: Cardiovascular Diseases
SMD: Standardized Mean Difference
PRISMA: Preferred Reporting Items for Systematic Reviews and Meta-Analyses
IQR: Interquartile Range

## STANDARDS OF REPORTING

PRISMA guidelines and methodology were followed.

PRISMA checklist is available on the publisher’s website.

## SUPPLEMENTARY MATERIAL

PRISMA checklist is available as supplementary material on the publisher’s website along with the published article.
